# Risk of pediatric inflammatory multi-system syndrome (PIMS or MIS-C) in pediatric patients with COVID-19 presenting with gastrointestinal symptoms

**DOI:** 10.3389/fped.2022.904793

**Published:** 2022-07-15

**Authors:** Carlos Mauricio Jaramillo-Esparza, Rodrigo Vázquez-Frias

**Affiliations:** ^1^Pediatric COVID-19 Care Unit, National Institute of Health Children's Hospital Infantil de México Federico Gómez, Mexico City, Mexico; ^2^Department of Gastroenterology and Nutrition, National Institute of Health Hospital Infantil de México Federico Gómez, Mexico City, Mexico

**Keywords:** SARS-CoV-2, COVID-19, pediatric inflammatory multisystem syndrome, gastrointestinal manifestations, children

## Abstract

**Introduction and objectives:**

Pediatric inflammatory multisystem syndrome (PIMS) is a life-threatening complication in pediatric patients with SARS-CoV-2 infection. An increase in the association of gastrointestinal symptoms and the presence of PIMS has been observed. The objective of this study was to analyze whether pediatric patients with COVID-19, who debut with gastrointestinal symptoms, have a higher risk of developing PIMS.

**Material and methods:**

An observational, analytical and retrolective study was carried out with a review of the records of patients diagnosed with COVID-19. Demographic, clinical and laboratory variables were recorded.

**Results:**

A total of 248 patients who met the selection criteria were included. Of Those 40% were female, with a mean age of 7 +/- 5.8 years. Gastrointestinal symptoms were the initial presentation in 103 patients, with vomiting being the most frequent symptom, followed by abdominal pain and diarrhea. In total 52 patients developed PIMS, 30 of whom presented with gastrointestinal symptoms. A RR of 1.57 (97% CI of 1.17–2.11) was found for the presentation of PIMS in patients positive for SARS-CoV-2 who present with gastrointestinal symptoms.

**Conclusions:**

There is an increased risk of developing pediatric multisystem inflammatory syndrome when there are gastrointestinal symptoms in pediatric patients with COVID-19.

## Introduction

SARS-CoV-2 infection has spread throughout the world and affects patients of all ages. The proportion of symptomatic pediatric patients is lower than that of the adult population. Until recently it was thought that children had a short course of the disease without complications; however, a group of children with severe multisystem disease known as pediatric inflammatory multisystem syndrome (PIMS or MIS-C), characterized by fever and multiple organ dysfunction were found to be associated with infection SARS-CoV-2 and development of COVID-19 (1). In addition, an increased frequency of gastrointestinal symptoms has been observed in MIS-C, which could potentially confuse the diagnosis of MIS-C with that of other gastrointestinal conditions, both infectious and inflammatory (2). In the first article published on the subject in the United Kingdom, a case series with 8 pediatric patients with MIS-C was described, finding a total of 100% of pediatric patients with gastrointestinal (GI) symptoms (3). Similarly, another study conducted in Italy described a series of cases with 10 patients with the same characteristics, finding 60% of patients with GI symptoms (4). This contrasts with studies done in adults, where <15% of cases with gastrointestinal symptoms are reported (5, 6). The objective of this study is to analyze whether pediatric patients with COVID-19 presenting with gastrointestinal (GI) symptoms had a higher risk of presenting pediatric multisystem inflammatory syndrome compared to those without GI symptoms.

## Materials and methods

A retrospective and retrolective study was carried out to review the records of patients with a proven diagnosis of COVID-19 who required hospitalization at *Hospital Infantil de México Federico Gómez* (HIMFG) from April 27th, 2020, to May 9th, 2021. All pediatric patients who had a positive test for SARS-CoV-2 (RT- PCR test for SARS-CoV-2, by QUANT STUDIO 5 applied biosystems by Thermo Fisher equipment) were included, those patients who did not have laboratory results for acute phase reactants during their hospitalization were excluded. PIMS/MIS-C was defined according to the CDC criteria, which include patients under 21 years of age with fever >38.0, with involvement of two or more organs or systems, with elevated acute phase reactants (ESR/CRP/Ferritin); which were determined as high according to the values established by the hospital laboratory (HIMFG); as well as not having another disease that explains the patient's symptoms (7, 8). We considered the following laboratory studies as APR (as established by CDC guidelines): C-Reactive protein (CRP), Ferritin and D-Dimer; which were determined as high according to the values established by our institution, as follows: CRP > 0.3 mg/dl, Ferritin > 290 μg/L and D-Dimer > 550 ng/mL. A positive gastrointestinal symptom was defined as those patients who presented any of the following symptoms on admission: diarrhea, vomiting, or abdominal pain. Those with hyporexia, nausea or constipation were excluded because they were not considered within the PIMS/MIS-C spectrum (9). In order to identify every patient with/without PIMS/MIS-C a thorough search through patient's files was conducted. With the objective of determining if every patient fulfilled the criteria established for PIMS/MIS-C and determine the cause of admission, as well as the presence of other factors that could influence or discard the diagnosis of PIMS/MISC as according to CDC criteria, excluding those patients with initial suspicion of PIMS/MIS-C that had other diseases that explained the symptoms at admission or during hospital stay. In accordance with international literature we considered PIMS diagnosis up to 6 weeks after SARS-CoV-2 detection (9).

All data were analyzed using IBM SPSS Statistics for Windows, Version 21.0 (IBM Corp., Armonk, NY, USA). Normally distributed variables are summarized as the mean and standard deviation, data from skewed distributions are shown as the median (range), and categorical variables are summarized as frequency and percentages. A (two-tailed) *P*-value < 0.05 was considered to be significant. Odds ratios (ORs) with 95% confidence intervals (CIs) were computed for significant categorical variables. Predictive factors for MIS-C were identified using a multiple logistic regression model including variables that were significant in univariate analysis. A value of *P* < 0.05 was considered as significant. Given the retrospective nature of the study, informed consent was not required.

## Results

A total of 278 patient records were reviewed, excluding 30 files that didn't complete our inclusion criteria. Ultimately 248 patients were included in the study. All of them had a positive PCR test for SARS-CoV-2 (we didn't identified the strain). Of these 113 were female (40%), with a mean age of 7 years (SD: +/- 5.76). The main demographic data can be found in Table 1. Gastrointestinal symptoms were the main presentation of 103 patients (40%); of these, the most frequent symptom was vomiting (*n* = 57; 55%), followed by abdominal pain (*n* = 53; 51%) and diarrhea (*n* = 30; 29%). In addition, 41 patients (39%) presented more than one gastrointestinal symptom at the time of admission.

**Table 1:** Demographics, gastrointestinal presenting symptoms and associated comorbidities, and laboratory values.

	**PIMS/MIS-C**		***p*** **-value**
	**Yes (*****n*** = **52)**	**No (*****n*** = **196)**	
**Age, years (Median +/- SD)**	**8.8 ± 6.1**	**7.5 ± 5.6**	**0.260**
**Sex Female, (%)**	**48**	**45**	**0.682**
**Gastrointestinal symptoms (%)**	**30 (57.6 %)**	**72 (36.7 %)**	**0.006**
**Comorbidities *n* (%)**	**30, (58%)**	**169 (86%)**	**0.000**
**Surgery**	**22 (73%)**	**25 (15%)**	
**Gastrointestinal**	**1 (3%)**	**13 (7%)**	
**Renal**	**5 (16%)**	**19 (11%)**	
**Neurological**	**3 (10%)**	**23 (13%)**	
**Hematological**	**5 (16%)**	**43 (25%)**	
**Cardiological**	**2 (6%)**	**8 (5%)**	
**Immunological**	**4 (13%)**	**1 (1%)**	
**Infectological**	**0 (0%)**	**2 (1%)**	
**Oncologic**	**3 (10%)**	**20 (11%)**	
**Pulmonary**	**1 (3%)**	**5 (3%)**	
**Endocrinological**	**4 (13%)**	**10 (6%)**	
**Hemoglobin g/dl (Mean ±SD)**	**12.4 ± 3.0**	**12.2 ±- 2.7**	**0.334**
**White blood cells 10^**3**^/mL (Mean ±SD)**	**11,600 ± 8147.7**	**9887.08 ± 13603.42**	**0.841**
**Lymphocyte count 103/mL (Mean + SD)**	**2460.3 ± 2138.3**	**2747.6 ± 2920.6**	**0.441**
**Platelet count 103/mL (Mean ±SD)**	**222,000 ± 128,301**	**232,000 ± 155,070**	**0.087**
**CRP mg/dl (Mean + SD)**	**6.78 ± 7.68**	**5.82 ± 7.22**	**0.552**
**Procalcitonin ng/mL (Mean + SD)**	**4.06 ± 16.5**	**1.55 ± 5.23**	**0.009**
**Ferritin μg/L (Mean + SD)**	**928.2 ± 2282.4**	**1080.5 ± 3990.1**	**0.647**
**D-dimer ng/mL (Mean + SD)**	**32.8 ± 23.7**	**39.9 ± 26.2**	**0.085**
**Fibrinogen (Mean + SD)**	**421.9 ± 170.1**	**444.5 ± 186.5**	**0.442**
**Creatinine mg/dl (Mean + SD)**	**1.13 ± 2.4**	**1.45 ± 3.17**	**0.234**
**Albumin g/dL (Mean + SD)**	**3.12 ± 0.83**	**3.28 ± 0.69**	**0.278**

A total of 52 patients (21%) met the criteria established by the CDC for PIMS/MIS-C during their hospitalization, observing 16 patients (30%) with PIMS/MIS-C diagnosis upon admission and 36 patients (70%) that developed PIM/MIS-C during hospitalization; of these, 30 patients (57%) debuted with gastrointestinal symptoms. Univariate and multivariate logistic regression analysis revealed that patients with positive RT-PCR for SARS-CoV-2 who debut with gastrointestinal symptoms (OR 2.35; 97% CI of 1.26–4.37) were significantly associated with PIMS/MIS-C. It is worth mentioning that no deaths or other adverse events were found among the patients studied. The comparison of patient's characteristics, comorbidities and biometric laboratory results can be found in Table 1.

Cause of admission was variable. Most patients were admitted due to complications of previous illness; COVID-19 related complications was the second most common cause of admission. With 68 patients (27.4%) admitted due to hematologic/oncologic related complications (e.g., neutropenic fever). While 54 patients (21.7%) were admitted because of COVID-19 related illness, having 16 patients admitted due to suspicion of PIMS/MIS-C (29%) and 38 patients (71%) admitted due to COVID-19 pneumonia. Other causes of admission were surgical related (appendicitis, volvulus, etc.) in 28 patients (11.2%), previous renal disease related in 20 patients (8%), cardiac disease decompensation in 9 patients (3.6%), and other less frequent (i.e., foreign body ingestion, moderate head trauma, etc.). The diagnosis of some surgical cause at admission was associated with a lower risk of developing PIMS regardless of having or not having gastro-intestinal manifestations.

Although it wasn't possible to determine the strain of SARS-CoV-2 in our institution (HIMFG) until after our study was finished (October 2021), based on epidemiological data we can assume our patients were infected with the Delta strain of the SARS-CoV-2 virus.

## Discussion

At the start of the COVID-19 pandemic very little was known about the SARS-CoV-2 virus, little by little we have gained new knowledge to answer the questions regarding this new virus, however, as the knowledge about this disease increases, the more questions we have. COVID-19 severe disease and associated mortality has been frequent in older adults and in patients with comorbidities, such as: diabetes mellitus, cardiovascular disease, chronic lung disease, obesity. All of which are risk factors that were identified from very early stages of the pandemic for higher morbidity and mortality, even for readmission (10–12). Some of these factors are shared in the pediatric population, in which obesity, diabetes, heart disease, chronic lung disease, epilepsy, and an immunocompromised state are also known to pose a higher risk of morbidity and mortality (13). Initially, it was not clear whether gastrointestinal symptoms were part of the spectrum of the disease, as such they were not even considered in the first reports. Furthermore, it was shown that these symptoms are present in both adult and pediatric patients with COVID-19, and even how they can appear even before the typical respiratory manifestations of the disease (prodromes) appear (14–16). Gastrointestinal symptoms are non-specific: nausea, vomiting, anorexia, abdominal pain, diarrhea, and even gastrointestinal bleeding, as well as elevated aminotransferases, amylase and lipase, and SARS-CoV-2 can even injure the endocrine pancreas (16). It was also hypothesized whether fecal-oral transmission was feasible, which has now been excluded (17). For a long time, there was speculation about whether the presence of these gastrointestinal manifestations meant a greater risk of having a more severe course of the disease, with sometimes controversial and marginal results (15, 18, 19).

In general, it is considered that SARS Cov2 infection in the pediatric population is usually asymptomatic or has few symptoms and for that the true prevalence of infection in children is not known (20). However, there are cases in which a potentially serious disease develops, such is the case of PIMS/MIS-C, an entity still not well known, that took the medical community by surprise. Acute gastrointestinal symptoms (diarrhea, vomiting or abdominal pain) are one of the criteria that patients may have to confirm the diagnosis of PIMS-MIS-C as established by the CDC and WHO, in accordance with international criteria. In a systematic review of children and adolescents with PIMS, it was shown that up to 71% of patients had gastrointestinal manifestations, including abdominal pain in 34% and diarrhea in 27% (21). Another systematic review, which included 27 studies corresponding to 917 cases of children and adolescents with PIMS, showed that gastrointestinal manifestations occur in up to 87.3% (95% CI 82.9–91.6).

Even though only few studies have described the presentation of gastrointestinal symptoms initially associated with PIMS/MIS-C, their results agree with ours, finding a high incidence of gastrointestinal symptoms in patients positive for SARS-CoV-2 who present or develop subsequently PIMS/MIS-C (2, 22), supporting our hypothesis of higher risk of developing PIMS-MIS-C in the presence of gastrointestinal symptoms in hospitalized patients with COVID-19. Identification of PIMS is crucial as it can result in serious organ dysfunction, including myocardial dysfunction, which has a high mortality rate (23, 24). The interval between the infection with SARS-CoV-2 virus and the development of PIMS (usually between 2 and 4 weeks) may be the result of the immune response to the virus itself. According to data previously obtained, we can observe that there is an increased risk of developing PIMS/MIS-C in patients with SARS-CoV-2 infection who present GI symptoms in comparison to those who don't. Therefore, patients who present with GI symptoms and COVID-19 most be closely monitored because of the higher risk of developing PIMS/MIS-C (even in outpatients).

Although there is not enough evidence to prove that outpatients with GI symptoms are at higher risk of developing PIMS/MIS-C, we do recommend keeping them under observation, being the general recommendation to continue with constant communication and suggest follow-up visits or serial laboratory tests, especially during the sub-acute period of the disease, which is when PIMS/MIS-C mostly develops.

We also found that procalcitonin levels were higher in those who developed PIMS. A recent study indicates that a rise in PCT above 0.2 ng/mL is associated with an elevated risk in all-cause mortality, the need for non-invasive ventilation, and a longer duration of mechanical ventilation (25). A meta-analysis shows that increased procalcitonin values are associated with a nearly 5-fold higher risk of severe SARS-CoV-2 infection (OR, 4.76; 95% CI, 2.74–8.29). The heterogeneity among the different studies was found to be modest (i.e., 34%) (26). It is hypothesized that serial procalcitonin measurement may play a role for predicting evolution toward a more severe form of disease. We didn't find differences in other laboratories, as shows in other studies, were D-dimer levels were higher (27). Although commonly used APR (CRP, D-Dimer, Ferritin) did not have a *P*-value of statistical significance to correlate with PIMS/MIS-C; it should not be interpreted as a lack of utility for the diagnosis of PIMS, but rather as the criteria established for the diagnosis itself suggests, the elevation alone of this biometric factors does not correlate with the diagnosis of PIMS/MIS-C. Therefore, always consider the whole criteria (clinical, laboratory and lack of plausible diagnosis) for said diagnosis.

As we have stated before, even though GI symptoms and elevation of procalcitonin have positive statistical significance, both factors alone cannot establish the diagnosis of PIMS, however, both variables can be used to establish risk assessment in order to determine which patients require close supervision in a hospital setting.

It is important to mention that even though our institution is a national reference center for the management of pediatric patients with COVID-19, in addition to the fact that many of the patients of our pediatric population had comorbidities typical of a highly specialized hospital, a considerable proportion of the pediatric population with COVID-19 (especially those without comorbidities) are treated elsewhere. Therefore, the results obtained cannot necessarily be extrapolated to the entire pediatric population.

In conclusion, PIMS/MIS-C is a serious and potentially fatal complication of SARS-CoV-2 infection that occurs in the pediatric population. Based on our results where we observed an association between the presence of gastrointestinal (GI) symptoms in pediatric patients with COVID-19 and the subsequent development of PIMS/MIS-C, we can conclude that our hypothesis of a higher risk of developing PIMS-MIS-C in children with COVID-19 and GI symptoms is correct.

## Data availability statement

The original contributions presented in the study are included in the article/supplementary material, further inquiries can be directed to the corresponding author.

## Author contributions

CJ-E participated with the conception or design of the work, data collection, data analysis and interpretation, drafting the article, and final approval of the version to be published. RV-F participated with the design of the work, data analysis and interpretation, drafting the article, critical revision of the article, and final approval of the version to be published.

## Conflict of interest

The authors declare that the research was conducted in the absence of any commercial or financial relationships that could be construed as a potential conflict of interest.

## Publisher's note

All claims expressed in this article are solely those of the authors and do not necessarily represent those of their affiliated organizations, or those of the publisher, the editors and the reviewers. Any product that may be evaluated in this article, or claim that may be made by its manufacturer, is not guaranteed or endorsed by the publisher.
